# A study of preparing silver iodide nanocolloid by electrical spark discharge method and its properties

**DOI:** 10.1038/s41598-021-99976-5

**Published:** 2021-10-14

**Authors:** Kuo-Hsiung Tseng, Chu-Ti Yeh, Meng-Yun Chung, Yur-Shan Lin, Ning Qui

**Affiliations:** 1 Department of Electrical Engineering, National Taipei University of Technology, Taipei, 10608 Taiwan, ROC; 2 Innolux Corporation., Southern Taiwan Science Park, Kaohsiung City, 82151 Taiwan, ROC

**Keywords:** Nanoscale materials, Nanoparticles, Structural properties

## Abstract

This study employed an electric discharge machine (EDM) and the Electrical Spark Discharge Method (ESDM) to prepare silver iodide nanocolloid (AgINC). Povidone–iodine (PVP-I) was dissolved in deionized water to create a dielectric fluid. Silver material was melted using the high temperature generated by an electric arc, and the peeled-off material was reacted with PVP-I to form AgI nanoparticles (AgINPs). Six discharge pulse wave parameter combinations (Ton–Toff) were employed, and the resultant particle size and suspension of the prepared samples were examined. The results revealed that AgINPs were successfully created using the ESDM. When Ton–Toff was set at 90–90 μs, the zeta potential of the AgINC was − 50.3 mV, indicating excellent suspension stability. The AgINC particle size was 16 nm, verifying that the parameters yielded AgINPs with the smallest particle size distribution and highest zeta potential. Ultraviolet–visible spectrum analyser was performed to analyse the samples, and the spectra indicated that the characteristic wavelength was 420 nm regardless of the Ton–Toff values. X-ray diffraction analysis determined that the AgINPs exhibited two crystal structures, namely β-AgI and Ag. Transmission electron microscopy was performed and revealed that the particles were irregularly shaped and that some of the larger particles had aggregated. The crystal structure was determined to be a mixture of Ag and β-AgI, with a lattice spacing of 0.235 nm and 0.229 nm, respectively. The lattice spacing of the Ag was 0.235 nm. X-ray diffraction analysis indicated that the prepared AgINC were composed of only Ag and I; no additional chemical elements were detected.

## Introduction

Nanomaterials are crucial in the development and application of nanotechnology^1^. Various effects occur when the size of a particle is reduced, such as small size, surface, quantum size, and quantum tunnelling effects. These effects cause materials to exhibit various macroscopic characteristics. Nano silver iodide (AgI) has various applications, including in photocatalysis, sensors, and fast ionic conductors^2–5^. For example, polytype AgI nanoplates enable successful battery operation at room temperature^6^. AgI is an essential material with a crystal structure similar to that of ice; hence, it is often used to induce cloud seeding^7,8^. The unique photoelectrical properties and excellent conductivity of AgI render it a material with high potential in commercial applications^9,10^.

Current methods for preparing nano AgI are precipitation reaction, laser ablation, reverse microemulsion, and ultrasonic spray pyrolysis^11–14^. Although the precipitation reaction method is simple and inexpensive, particles aggregate easily. The ultrasonic spray pyrolysis method can be used to prepare particles with nanostructures. A liquid-phase substance is used as the precursor in ultrasonic spray pyrolysis, which yields highly pure and dispersed products. Nevertheless, this method requires the use of costly materials and considerable energy^15^.

In this study, an electrical discharge method similar to the submerged arc technique^16^ was used to prepare nanoparticles (NPs). This processing technique is called the Electrical Spark Discharge Method^17^ (ESDM). Both methods use two electrodes as workpieces, and an electric arc is generated to process the electrode materials. The workpieces are considered processors or processed objects according to user need. The submerged arc technique must be performed in a vacuum chamber while maintaining the dielectric fluid at a low temperature. By contrast, the ESDM does not entail a vacuum environment and requires only a dielectric fluid at normal temperature and normal pressure. The principle of the spark discharge generation technique^18^ is also quite similar to that of the method used in this study; however, it is mainly used to prepare aerosols, whereas the products examined in this study were colloids.

The ESDM preparation process does not require additional chemical agents or dispersants to generate nanocolloids (NCs) with favourable characteristics. The process is simple, fast, and environmentally friendly and reduces the likelihood of harming the human body or polluting the environment. In this study, six pulse wave parameter values (Ton–Toff) were configured using an electric discharge machine (EDM), and the different Ton–Toff values were used to prepare AgINC through the ESDM method. Next, the characteristics of the AgINC were analysed using precision instruments. Finally, dynamic light scattering (DLS) analysis was performed to determine which Ton–Toff value produced AgINC with superior characteristics and could thus serve as the most suitable parameter for AgINC preparation.

## Materials and methods

### Principle of electrical spark discharge method (ESDM)

**Figure 1:**
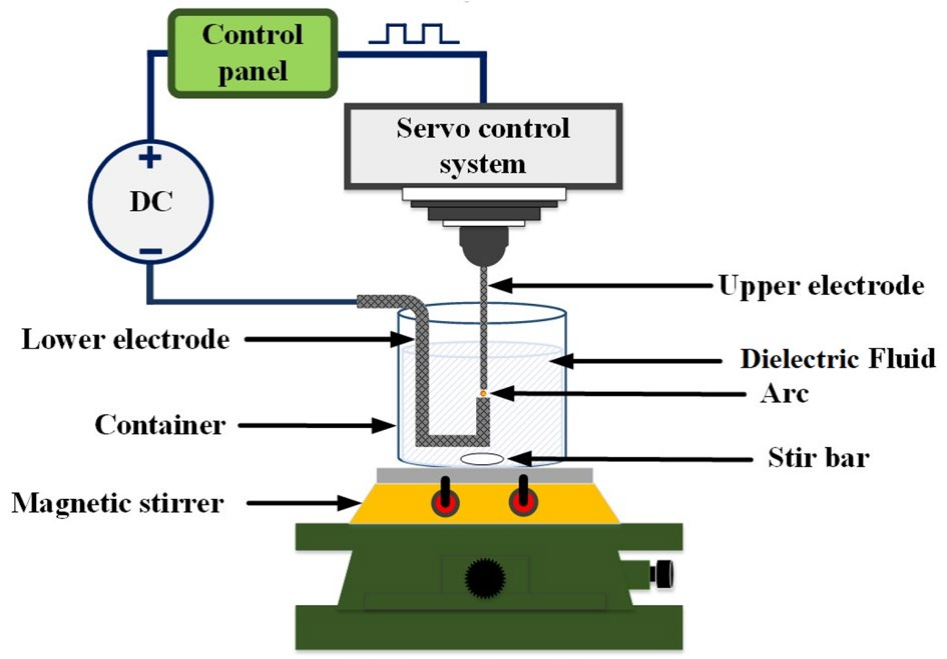
Schematic of an EDM.

In the ESDM, electrical energy is converted into thermal energy. The method is a thermal processing technique in which electrodes are used to melt materials quickly. The upper and lower electrodes are composed of conductive materials and are not in direct contact; hence, no physical force is generated. Figure 1 depicts the configuration of an EDM. The upper and lower electrodes are placed in a highly insulating dielectric fluid, and a direct current pulse from tens to hundreds of volts is produced between the electrodes^19^. A servo control system is used to control the upper electrode, which slowly approaches the lower electrode. When the two electrodes are sufficiently close, the electrical field strength between the small tips of the electrodes surpasses the insulation strength of the dielectric fluid. This leads to an electrical breakdown and generates a spark between the two electrodes, inducing an discharge^20^. The resulting high temperature can melt or even vaporize the electrodes, creating small particles that randomly disperse. The particles that enter the dielectric fluid are then rapidly cooled and consolidated to form NPs. Finally, as the Ton–Toff cycle ends, the surfaces of the two electrodes are cooled by the dielectric fluid, and the insulation is recovered until the next Ton–Toff cycle begins. The repeated cycle enables the preparation of NCs. Ton is the discharge pulse time. Because conservation of the electrode material is desired, the pulse duration might be extended, which expands the plasma region. This increase reduces the density of the plasma region, and the overall result is a reduction of effectiveness. Toff is the time between active pulse discharges. The electrodes require time to return to isolation status and the vaporized metallic particles time to cool and solidify. The discharge pulse wave parameter values (Ton–Toff) affect the accuracy of the electrical discharge machining and electrode consumption rate.

**Figure 2:**
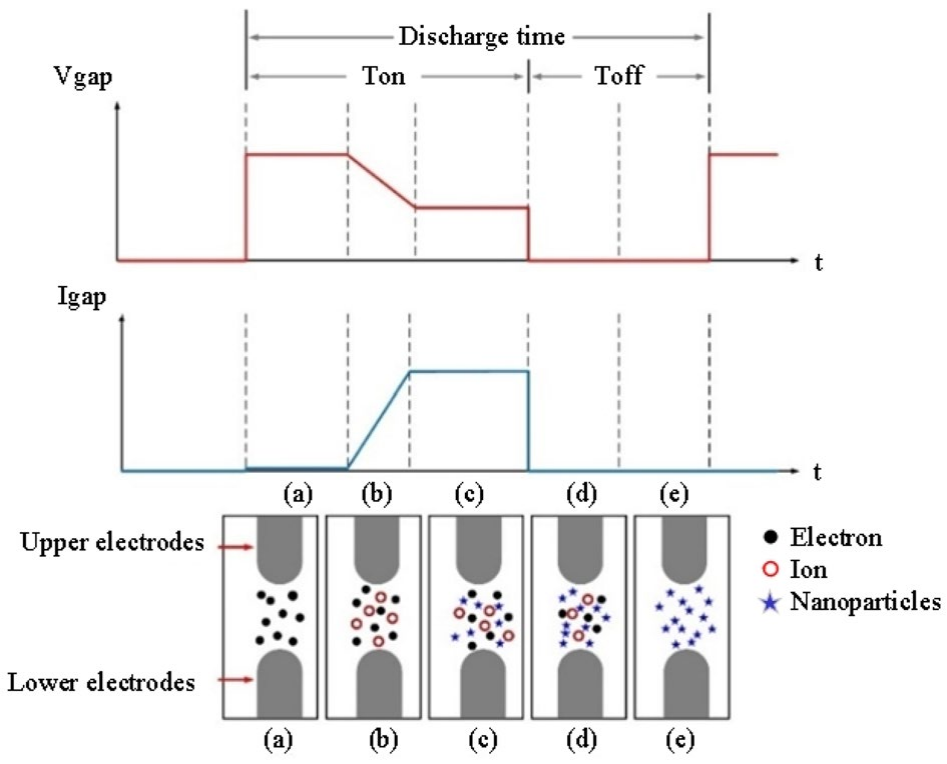
ESDM process: (**a**) voltage generation, (**b**) ionization effect, (**c**) melting effect, (**d**) termination of electric discharge, and (**e**) recovery of insulation.

Figure 2 details the ESDM process. Vgap and Igap represent the voltage and the current between the two electrodes, respectively. Ton and Toff respectively represent the time the current pulse is turned on and off^21,22^.

- Voltage generation: The upper and lower electrodes are submerged in dielectric fluid. A direct current source is connected to the two electrodes. When the electrical field strength at the tips surpasses the insulation strength of the dielectric fluid, electrons are ejected from the lower electrode to the upper electrode to form an electric arc with a temperature of 5000–6000 K^23^.
- Ionization effect: When an electrical breakdown occurs in the dielectric fluid, electrons are ejected from the lower electrodes; the valence electrons in the outermost shell of the atoms are excited to form cations. The electrons travel rapidly toward the upper electrode and reinduce the ionization effect to form an ionization channel^24^. This causes the current to increase and generate a gap current. At this moment, the gap is approximately 20–30 µm^25^.
- Melting effect: When these ions impact the metal electrode surface, kinetic energy is converted into thermal energy to form an electric arc, the high temperature of which causes the surface metal to detach and melt. Accordingly, metal particles are produced from the metal electrode surface.
- Termination of electric discharge: The ionization channel quickly disappears. The metal particles and ions generated during the melting process become suspended in the dielectric fluid.
- Recovery of insulation: When the surface temperature of the metal electrodes decreases, the insulation ability and pressure resistance of the dielectric fluid are recovered. Metal particles are scattered in the dielectric fluid.

### Preparation of AgINC using the ESDM

This study used the ESDM to prepare AgINC. Silver lines with 99.99% purity were used as electrodes. A total of 1 mL of povidone–iodine (PVP-I) was mixed with 199 mL of deionized water to prepare dielectric fluid. The fluid’s pH was 6.2 and its electrical conductivity 57 μS/cm. Parameters of the preparation environment are listed in Table 1. Different Ton–Toff parameters were employed to prepare the AgINC samples. The current segment setting was IP = 1 in level.

**Table 1:** EDM parameters for preparing AgINC.

Ton-Toff	10–10, 30–30, 50–50, 70–70, 90–90, 110–110 (µs)
Temperature	25 °C
Atmospheric pressure	1 atm
Material	Ag wire, 99.99%
Diameter of material	Upper electrode: 1 mm Lower electrode: 2 mm
Volume of dielectric fluid	200 ml
Dielectric fluid	Povidone-iodine (1 ml) + deionized water (199 ml)
Current segment setting	IP = 1 in level
Voltage	140 V
Discharge time	5 min

### Apparatus settings

An ultraviolet–visible spectrum analyser (UV–Vis, Thermo-Helios Omega, Thermo Fisher Scientific Inc., Waltham, MA, USA) was used to examine the optical properties of the AgINC. The start and stop wavelength were 190 and 600 nm, respectively. The scanning speed and interval were 240 nm/min and 1 nm, respectively. A zetasizer (Zetasizer Nano ZS90, Malvern Zetasizer, Worcestershire, UK) was used to obtain the suspension stability and particle size distribution. An absolute value of zeta potential over 30 mV indicated favourable suspension stability^26^. The light source of the zetasizer was a He–Ne laser (633 nm). The scattering angle to measure particle size was 90°. The dispersant setting was water at 25 °C, viscosity was 0.8872 cP, and refractive index was 1.330. X-ray diffraction (XRD) was used to analyse the crystal structure. The range of the 2θ was 10°–60°, step size was 0.05, and time per step was 0.5 s. The sample was in powder form. At varying pressure and temperature, AgI exhibits different crystal structures. The most researched structures are the α-, β-, and γ-phases. α-AgI exhibits a body-centred cubic crystal system; β-AgI has a wurtzite hexagonal crystal system; and γ-AgI exhibits a zincblende cubic crystal system. Under normal temperature and pressure, the stable crystal structures of AgI are the β- and γ-phases^27^. Therefore, AgI prepared using general methods usually contains a mixture of β- and γ-phases^28^. Transmission electron microscopy (TEM, JEM-2100F, JEOL Ltd., Japan) was used to analyse particle shape, size, and structure. The energy was as high as 200 kV, and the magnification was 10,000X, 40,000X, and 80,000X. An energy-dispersive X-ray spectroscopy (EDS, JEM-2100F, JEOL Ltd., Japan) instrument, which was attached to the TEM device, was used to conduct elemental analysis.

## Results

### Formation of AgINPs

**Figure 3:**
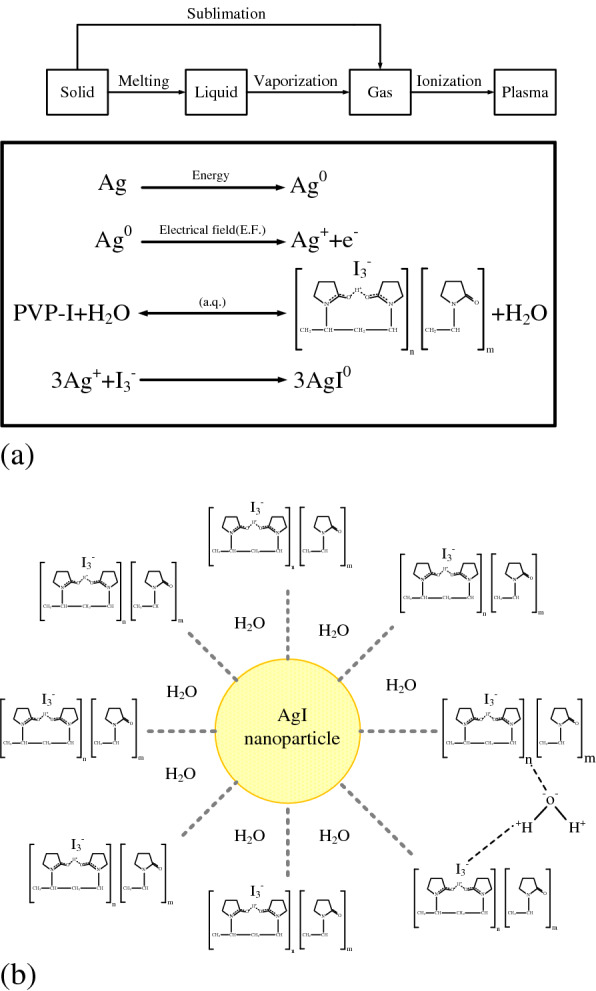
(**a**) Ionization of AgI; (**b**) suspension of AgINPs.

H_2_O and PVP-I was used as the dielectric fluid, which thus comprised H^+^ and OH^−^. During the discharge process, a high-energy spark was created to melt solid Ag, which was then progressively vaporized to form Ag atoms. Under the catalytic effect of the electrical field, the Ag atoms lost their electrons to form Ag^+^ ions. When the electrical field strength reached 500 kV/cm (the discharge gap was 20 μm, and the discharge voltage was approximately 100 V), the Ag atoms were decomposed into Ag^0^, Ag^+^, and e^−^ and reacted with I^3−^ and H_2_O to form AgI. OH^−^, which carries a negative charge, reacted with H_2_O to form colloids through hydrogen bonding. In detail, I was embedded in the PVP-I during the electrical field discharge. I^−^ dissociated and became the structure PVP^+^…I^−^. During this period, Ag^0^ dissociated into Ag^+^ and e^−^ under the electrical field; thus, Ag^+^ and I^−^ combined to form AgI, and PVP^+^ and e^−^ were reduced to PVP^0^. Simultaneously, the PVP^0^ coated the surface of AgI, which provided protection for the AgI, and the OH groups on the PVP were suspended in water by hydrogen bonds. Figure 3(a) illustrates the ionization process of the AgI and Fig. 3(b) the mechanism of AgINP suspension.

### Zetasizer and UV–Vis results of AgINC prepared using the ESDM

**Figure 4:**
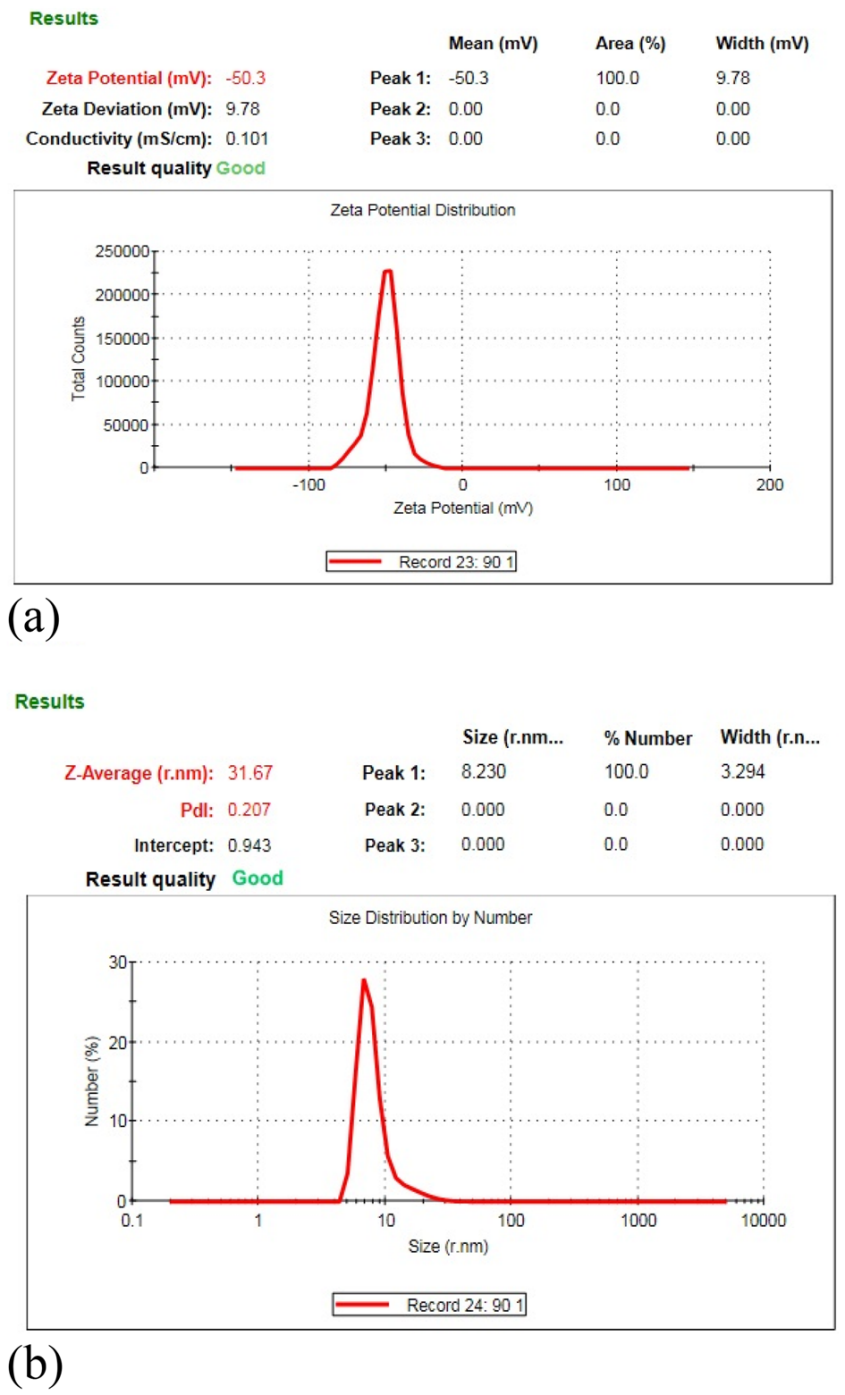
AgINC (**a**) zeta potential and (**b**) size distribution.

The sample used in the analysis was prepared using Ton–Toff = 90–90 μs. Figure 4(a) presents the zeta potential of the AgINC sample created using the optimal parameter setting. The zeta potential reached − 50.3 mV, indicating that the sample exhibited outstanding suspension stability. Figure 4(b) presents the particle size distribution of the sample. The most common particle size was 16 nm (radius = 8 nm).

**Figure 5:**
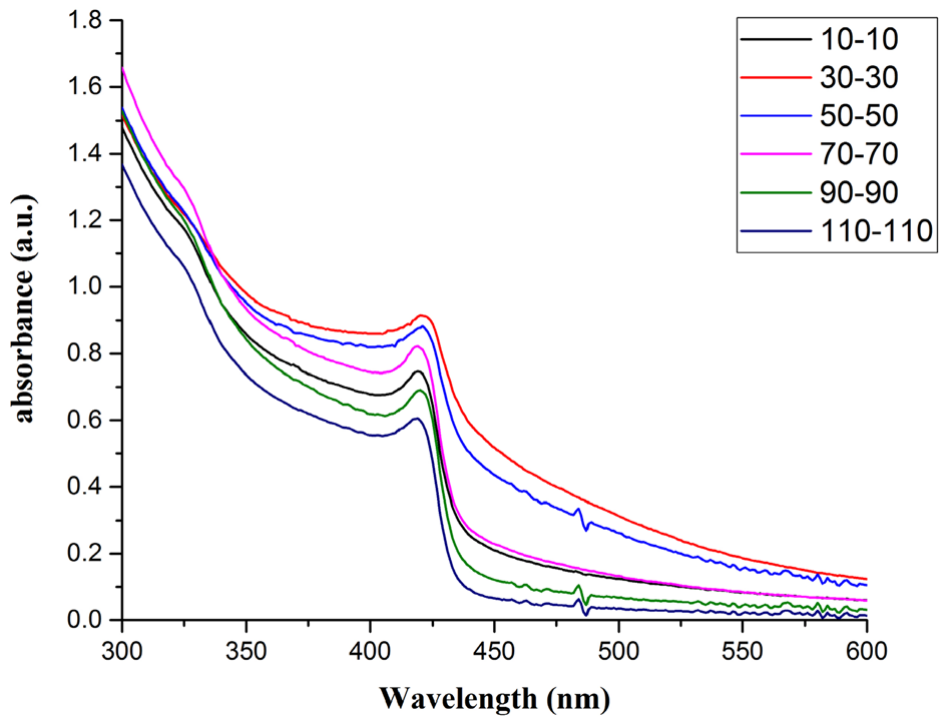
UV–Vis analysis results.

Figure 5 presents the UV–Vis analysis results for the AgINP samples. The results revealed that the characteristic wavelength was 420 nm for all AgINC samples regardless of the Ton–Toff values. The position of the absorption peak will be displayed at the wavelength where the particle size accounts for the largest proportion and change due to the difference of particle size. AgINPs prepared by ESDM have a large size range which makes the peak not shifting. Ton–Toff = 30–30 μs yielded the largest absorbance value (0.914), whereas 110–100 μs yielded the smallest absorbance value (0.605).

Table 2 compiles the analysis results acquired using UV–Vis and the zetasizer. The Z-average is the average particle size obtained using the cumulants technique in DLS and is based on the scattered light intensity (%). Size (r.nm) is determined by converting the intensity (%) into a number (%), with the particle number used as a reference to estimate the particle size. The polydispersity index (PDI) is the distribution coefficient used to describe the distribution of the polymer molecular weight or the distribution width of the particle size. Width refers to the distribution width. For the different Ton–Toff parameters, the peaks of the particle size distributions were all under 50 nm, indicating that most of the prepared samples were nanosized. The results indicated that the optimal zeta potential (− 50.3 mV) was achieved when Ton–Toff was 90–90 μs, showing the excellent suspension stability of the corresponding sample. For this parameter value, the PDI was 0.20, and the width was 3.2 nm (in radius). In consideration of both the particle size distribution and the zeta potential, the smallest particle distribution and the optimal stability suspension were achieved when Ton–Toff was 90–90 μs, which was thus the optimal preparation parameter setting.

**Table 2:** Results for absorbance, zeta potential, and particle size distribution.

Ton-Toff (μs)	Absorbance	Zeta potential (mV)	Size (r. nm)	Z-average (r. nm)	PDI	Width (r. nm)
10–10	0.747	− 43.0	18	38	0.27	5.6
30–30	0.914	− 46.4	22	39	0.24	6.3
50–50	0.883	− 47.4	23	39	0.21	6.5
70–70	0.822	− 46.6	17	31	0.24	5.0
90–90	0.691	− 50.3	8	31	0.20	3.2
110–110	0.605	− 42.3	12	29	0.23	3.9

### XRD and TEM of AgINC

**Figure 6:**
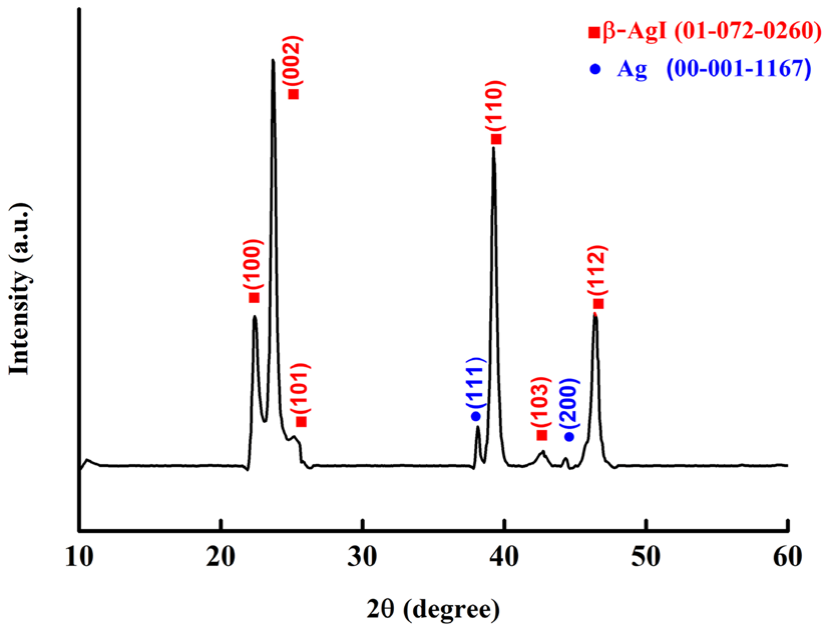
XRD analysis results.

Figure 6 presents the results of the XRD analysis. The AgINC sample was determined to consist of AgI (International Centre for Diffraction Data [ICDD]: 01–072-0260) and Ag (ICDD: 00–001-1167). The ICDD report of AgI suggested that the crystal system of this compound was hexagonal. This indicated that the crystal orientation of AgI was β, which differs from the crystal orientation of AgI synthesized through other methods. The Bragg angles of the β-AgI were 22.337°, 23.675°, 25.322°, 39.205°, 42.643°, and 46.313°, which respectively corresponded to the (100), (002), (101), (110), (103), and (112) crystal orientations. The Bragg angles of Ag were 38.1° and 44.4°, which corresponded to the (111) and (200) crystal orientations. The ICDD report suggested that Ag was present in the cubic system.

**Figure 7:**
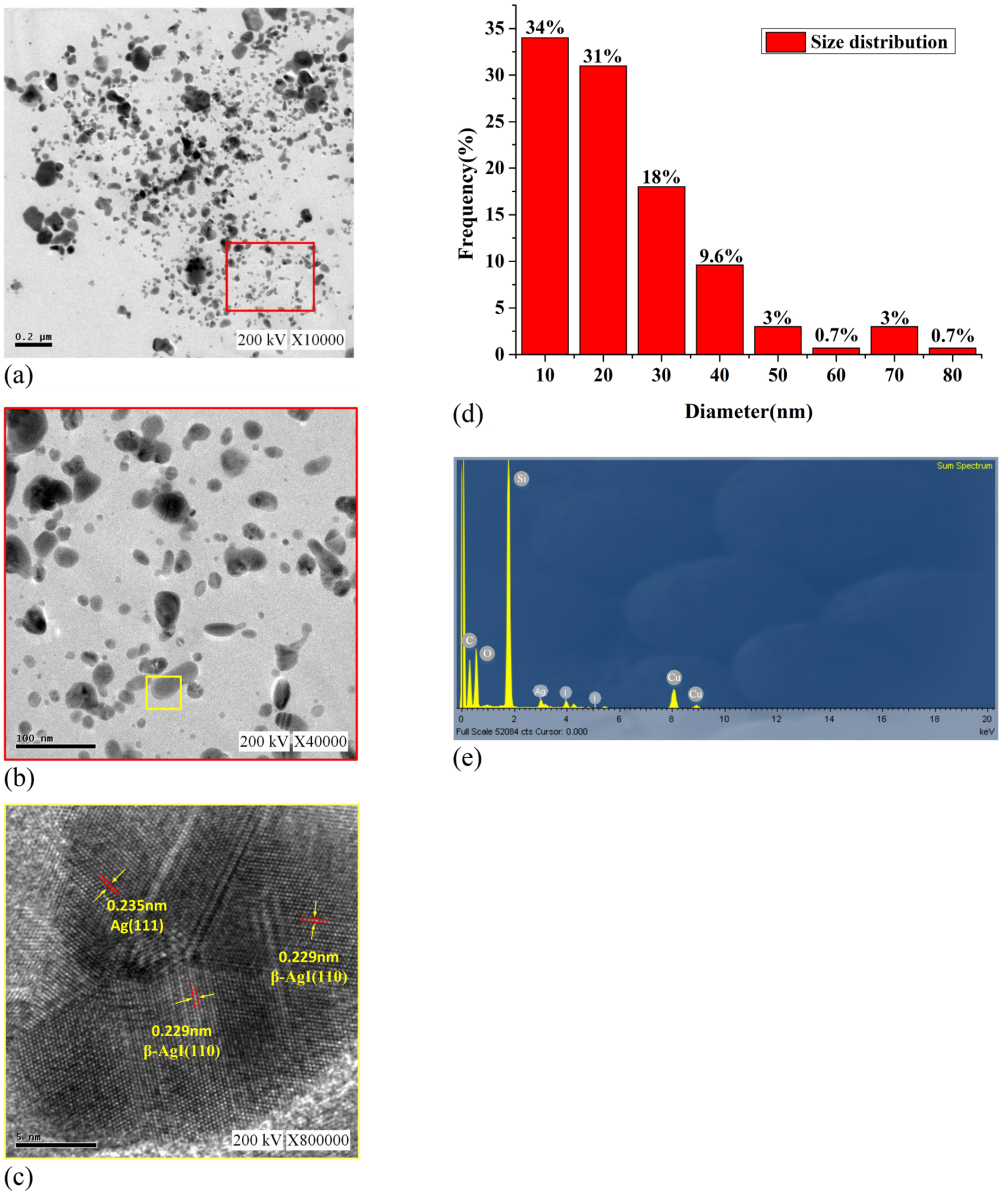
TEM analysis results at (**a**) 0.2 µm, (**b**) 100 nm, (**c**) 5 nm, (**d**) size distribution, and (**e**) EDS analysis.

Figure 7 presents the analysis results acquired through TEM and the EDS analyser attached to the TEM instrument (see the Supplementary Data). The samples is fabricated under 90–90 μs parameter. Figure 7(a) presents the image taken using a proportional scale of 0.2 µm. Affected by the Van der Waals force, it can be seen that the distance between small-sized AgINPs is larger, indicating better dispersion. On the contrary, the distance between large AgINPs is small, and some large particles are stacked and aggregate which indicates the poor dispersion.Fig. 7(b) presents the magnified image (proportional scale of 100 nm) of the area in the red frame in Fig. 7(a); this image indicates that the AgINPs had an irregular shape. An irregularly shaped particle was analysed using TEM. Figure 7(c) presents the magnified image (proportional scale of 5 nm) of the area within the yellow frame in Fig. 7(b); the image indicates that the lattice spacings of the β-AgI and Ag were 0.229 and 0.235 nm, respectively. Figure 7(d) presents the particle size statistics. The statistical results of Fig. 7(d) mainly comes from Fig. 7(b). The proportions of the AgINPs with a particle size of 0–10, 11–20, 21–30, 31–40, 41–50, 51–60, 61–70, and 71–80 nm were 34%, 31%, 18%, 9.6%, 3%, 0.7%, 3%, and 0.7%, respectively. Particles of size 0–10 nm were the most common. Figure 7(e) presents the elemental analysis results acquired using EDS. C represents organic matter, and O represents oxide. Si represents the silicon detector in the equipment, and Cu represents the substrate (copper net) used in the sample analysis. The results verified that the prepared AgINC sample did not contain any elements other than Ag and I. Table 3 provides the EDS analysis results of the AgINC. The mass fraction was 45.34 wt% for Ag and 54.66 wt% for I. The mole fraction was 49.39 at% for Ag and 50.61 at% for I. This indicated that the ratio of Ag and I in the prepared AgINPs was indeed 1:1, with no additional chemical elements present.

**Table 3:** EDS analysis of AgINC prepared using the ESDM.

Element	Peak area	Area sigma	K factor	Abs Corrn	Weight%	Sigma	Atomic%
Ag	15,803	391	1.724	1.000	45.34	0.75	49.39
I	17,491	304	1.877	1.000	54.66	0.75	50.61

### Discussion

The characteristics of the AgINC samples prepared using the ESDM were analysed using UV–Vis, EDS, XRD, and TEM to yield the following results.

1. Synthesis mechanism: Electric discharge machining was conducted on the Ag electrode in a dielectric fluid The high energy and high electrical field ionize Ag metal into Ag^+^, and the PVP-I in the dielectric fluid forms I_3_^−^. Next, Ag^+^ combines with I_3_^−^ in the dielectric fluid and generates AgI molecules. Finally, the AgI molecules form AgINPs that are suspended in the dielectric fluid and aggregate into AgINC.
2. Characteristic wavelength and absorbance: In the UV–Vis analysis, the characteristic wavelength of the prepared AgINPs was discovered to be 420 nm, which matched the optical characteristics of this particle. This indicated that all six Ton–Toff parameters were feasible for preparing AgINPs. However, the absorbance spectrum of the prepared AgINPs exhibited peaks that ranged from 0.605 to 0.914. This indicated that absorbance was indeed influenced by Ton–Toff. The six Ton–Toff parameters involve different discharge and cut-off times. When AgINC exhibited high absorbance, the discharge success rate in the manufacturing process was higher, but the product characteristics were not necessarily superior. The DLS technique was required for more precise evaluation of sample characteristics.
3. Suspension: The absolute zeta potential values of the AgINC samples prepared using the six Ton–Toff values were higher than 30 mV without any other dispersant being added. The samples exhibited favourable suspension stability. Ton–Toff = 90–90 μs yielded the optimal zeta potential (− 50.3 mV). This indicated that the AgINC prepared with this parameter setting had the greatest suspension stability and dispersibility. Thus, 90–90 μs is the most suitable parameter for the preparation of AgINC.
4. Particle size distribution: AgINPs were examined using DLS under the Number (%) condition. The size (r. nm) and z-average (r. nm) values of the six Ton–Toff parameters were lower than 50 nm. This indicated that the prepared products were nanosized. Comparison of the Ton–Toff parameters revealed that the smallest size (r. nm) of 8 nm was obtained when the parameters were 90–90 μs. Accordingly, the radius of most AgINPs in the AgINC was 8 nm with a z-average (r. nm) of 31.67 nm; the z-average is the average particle size of AgINPs after light intensity analysis. Ton–Toff = 90–90 μs was confirmed to be the most suitable combination for the preparation of AgINC with favourable characteristics according to the DLS analysis results.
5. XRD: The results suggested that the prepared AgINC were composed of β-AgI and Ag. Two possibilities exist for the presence of the Ag crystals. First, AgI is extremely photosensitive. The samples were prepared in the presence of indoor light, and XRD analysis entails applying a high-energy radiation beam to the sample surface. This caused the AgI to be reduced to Ag. Second, Ag crystals might have formed when the Ag^+^ ions in the colloid were not completely bound with I^−^ ions during the preparation process.
6. TEM: The sample prepared under 90–90 μs was subjected to TEM. The analysis revealed that most of the large particles had aggregated. Observation of the sample surface revealed that the AgINPs had irregular shapes. The lattice spacing of nanosilver was determined to be 0.235 nm, and that of AgINPs was 0.229 nm. These figures are consistent with those published by the ICDD. In the EDS analysis, the ratio of Ai to I in the AgINPs was nearly 1:1, with 49.39 at% Ag and 50.61 at% I. This verified that the prepared AgINPs contained no additional chemical elements.

## Conclusion

This study employed an EDM to perform the ESDM. PVP-I was dissolved in deionized water to create a dielectric fluid, and an electric arc was generated to melt the metal electrodes and thus prepare AgINC. The synthesis environment was maintained at normal pressure and temperature, and no additional chemical reagent except iodine was added. According to the study results, the following conclusions may be drawn.

The characteristic wavelengths of the AgINPs prepared with various Ton–Toff parameters were all 420 nm. Absorbance was affected by Ton–Toff. The AgINC prepared using Ton–Toff = 90–90 μs had the highest suspension stability (− 50.3 mV) and smallest particle size (8 nm). This indicates that 90–90 μs is the most suitable parameter combination for preparing AgINC. The characteristics of AgINC prepared at 90–90 μs were analysed using XRD and TEM. The XRD analysis results indicated that the AgINC had two crystal orientations, β-AgI and Ag. TEM analysis indicated that the particle size of most AgINPs was smaller than 20 nm. In addition, the EDS analysis revealed that the AgINC were composed only of Ag and I, with no other elements present in the product. In conclusion, the ESDM is a fast, simple, and environmentally friendly AgINC preparation method.

## Supplementary Information

Supplementary Information.

## Abbreviations

EDM: Electric discharge machine
ESDM: Electrical spark discharge method
AgINC: Silver iodide nanocolloids
PVP-I: Povidone–iodine
AgINPs: AgI nanoparticles
AgI: Nano silver iodide
NPs: Nanoparticles
NCs: Nanocolloids
DLS: Dynamic light scattering
UV–Vis: Ultraviolet–visible spectrum analyser
XRD: X-ray diffraction
TEM: Transmission electron microscope
EDS: Energy-dispersive X-ray spectroscopy
PDI: Polydispersity index
ICDD: International Centre for Diffraction Data
