# Supplementary material for: A study of preparing silver iodide nanocolloid by electrical spark discharge method and its properties
Source: Sci Rep. 2021 Oct 14;11:20457. doi: 10.1038/s41598-021-99976-5 (PMC8516939; doi:10.1038/s41598-021-99976-5)
Supplement: Supplementary file 1 — Supplementary Information. [file 41598_2021_99976_MOESM1_ESM.doc]

**A Study of Preparing Silver Iodide Nanocolloid by Electrical Spark Discharge Method and Its Properties**

Kuo-Hsiung Tseng^1*^, Chu-Ti Yeh^1^, Meng-Yun Chung^1^, Yur-Shan Lin^1^ and Ning Qui^2^

^1^ Department of Electrical Engineering, National Taipei University of Technology, Taipei, 10608, Taiwan, R.O.C.

^2^ Innolux Corporation., Kaohsiung City, Southern Taiwan Science Park, 82151, Taiwan, R.O.C.

*Correspondence should be addressed to Kuo-Hsiung Tseng:

**Abstract**

This study employed an electric discharge machine (EDM) and the Electrical Spark Discharge Method (ESDM) to prepare silver iodide nanocolloid (AgINC). Povidone–iodine (PVP-I) was dissolved in deionized water to create a dielectric fluid. Silver material was melted using the high temperature generated by an electric arc, and the peeled-off material was reacted with PVP-I to form AgI nanoparticles (AgINPs). Six discharge pulse wave parameter combinations (Ton–Toff) were employed, and the resultant particle size and suspension of the prepared samples were examined. The results revealed that AgINPs were successfully created using the ESDM. When Ton–Toff was set at 90–90 μs, the zeta potential of the AgINC was −50.3 mV, indicating excellent suspension stability. The AgINC particle size was 16 nm, verifying that the parameters yielded AgINPs with the smallest particle size distribution and highest zeta potential. Ultraviolet–visible spectrum analyser was performed to analyse the samples, and the spectra indicated that the characteristic wavelength was 420 nm regardless of the Ton–Toff values. X-ray diffraction analysis determined that the AgINPs exhibited two crystal structures, namely β-AgI and Ag. Transmission electron microscopy was performed and revealed that the particles were irregularly shaped and that some of the larger particles had aggregated. The crystal structure was determined to be a mixture of Ag and β-AgI, with a lattice spacing of 0.235 nm and 0.229 nm, respectively. The lattice spacing of the Ag was 0.235 nm. X-ray diffraction analysis indicated that the prepared AgINC were composed of only Ag and I; no additional chemical elements were detected.

**Keywords:** electrical spark discharge method, silver iodide nanocolloids, povidone–iodine

**Supplementary Data**

The paper gets the second round of experiment and send the sample to TEM to get the HRTEM image and Fast Fourier Transform (FFT) pattern. The results are shown in Supplementary Figure S1.


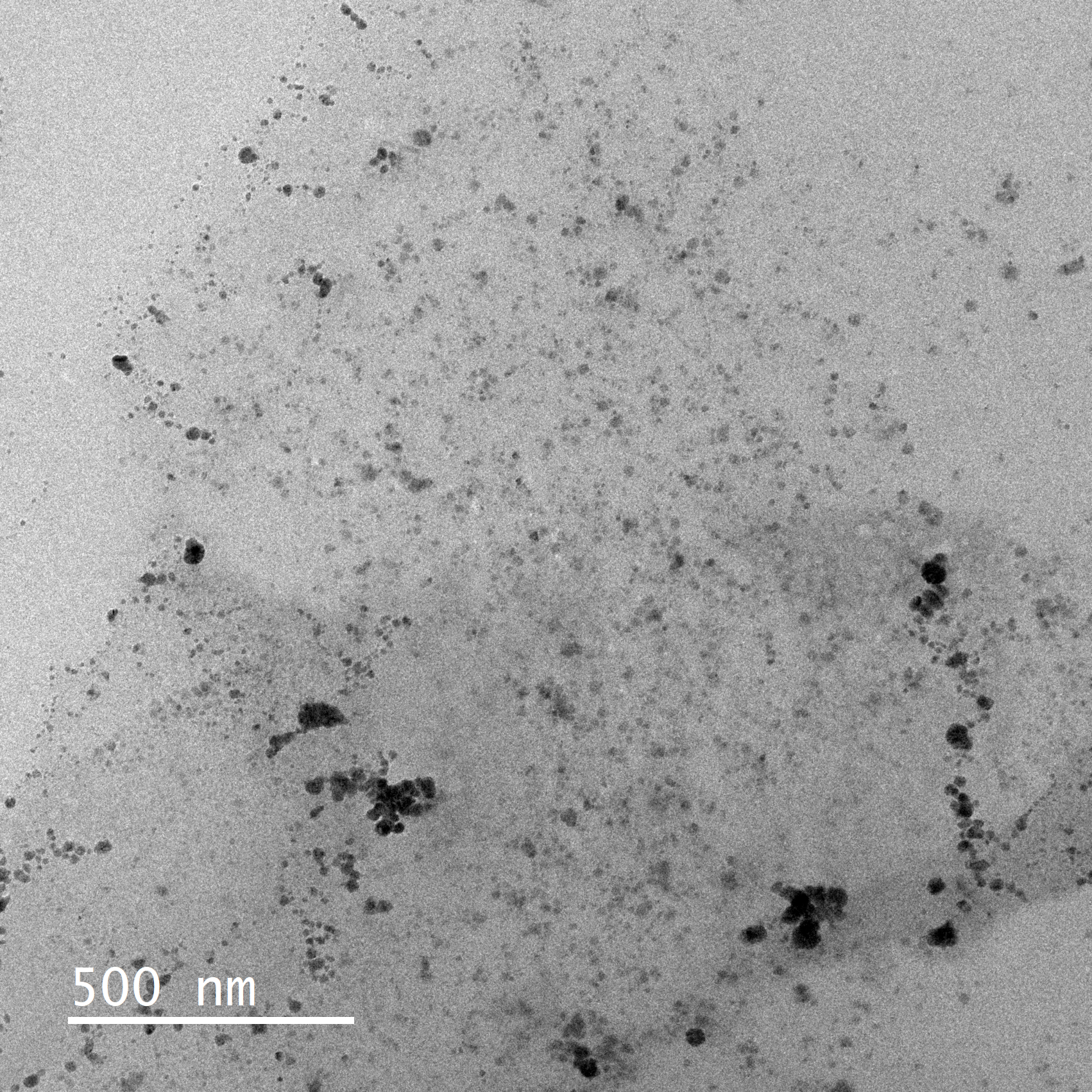

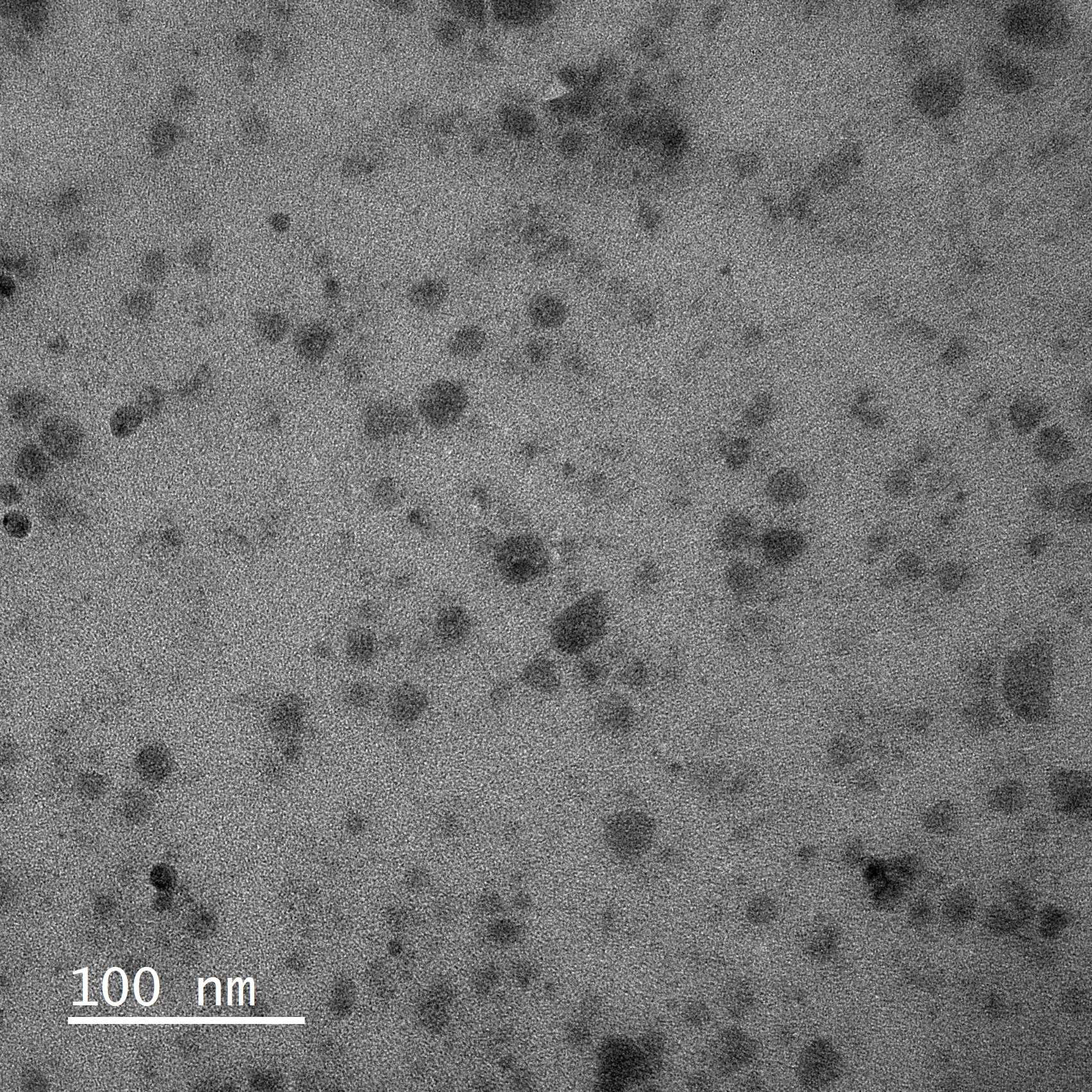


(a) (b)


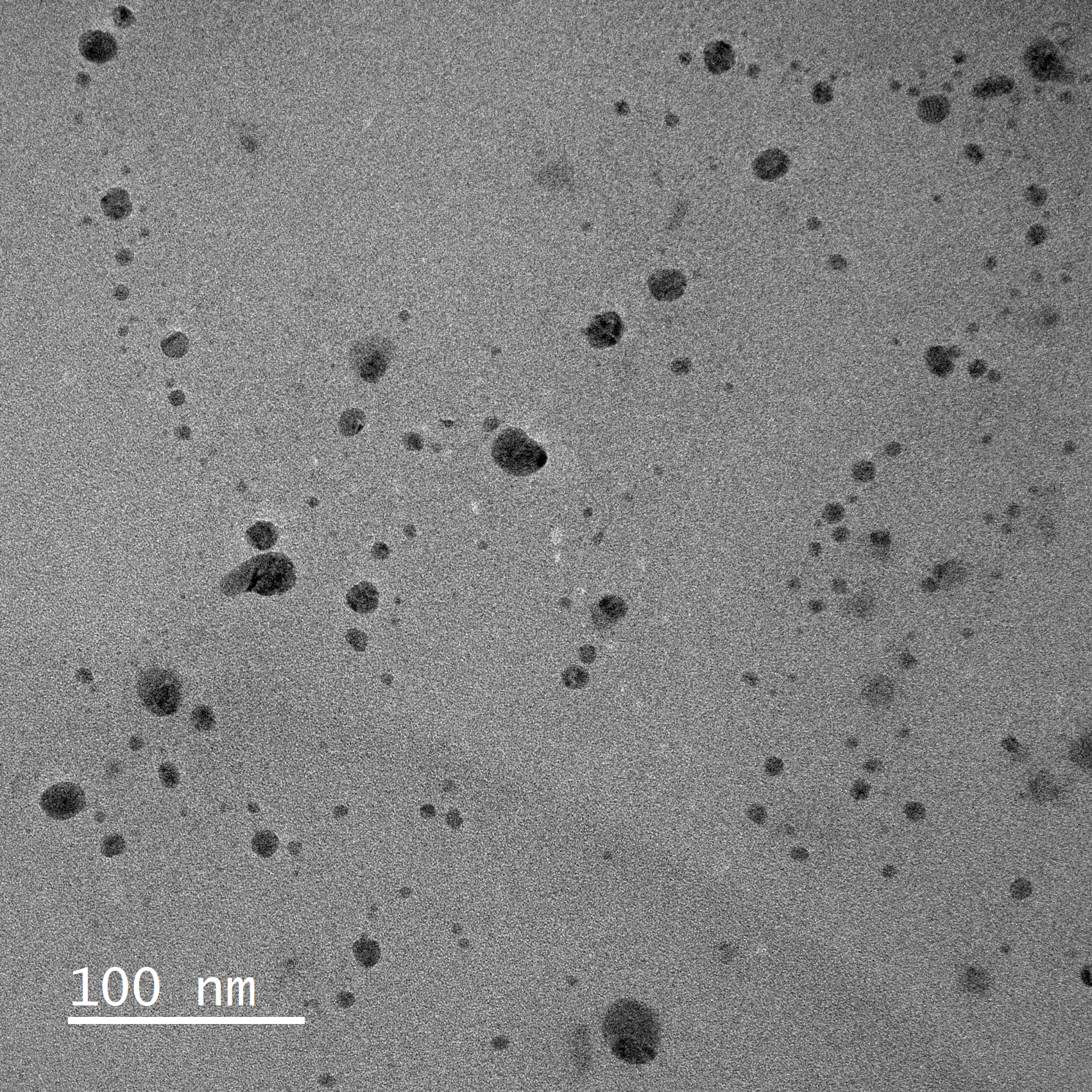

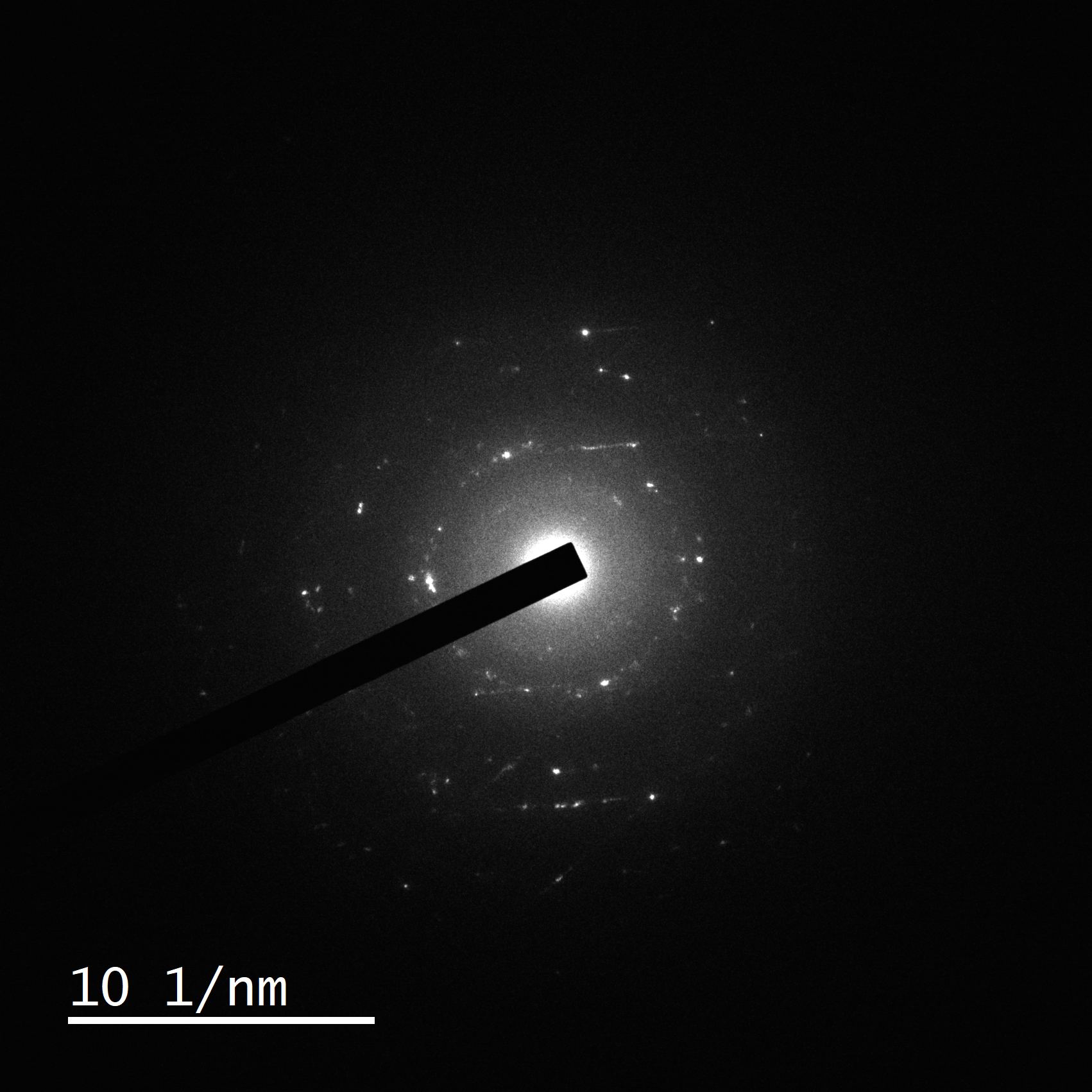


(c) (d)

Supplementary Figure S1 TEM analysis results at (a) 500 nm, (b) 100 nm, and (c) another image of 100 nm, and (d) FFT pattern.

Also, about the EDS mapping, the results are shown in Supplementary Figure S2. In this inspection process, a comprehensive scan is used. Because the AgINP particle size is too small, and the screen continue to produce a little shaking during the scanning process, both silver element (red) and iodine element (green) are distorted, Supplementary Figure S2 (b) and (c) separately, and the exact AgI phase cannot be clearly identified when scanning EDS mapping shown in Supplementary Figure S2 (d). The mass fraction of Ag in EDS is 39.61wt%, and I is 60.39wt%. In addition, the Ag of the mole fraction is 43.56at%, and the I is 56.44at%.

Due to the above reasons, the analysis switched to EDS scanning by taking points, as shown in Supplementary Figure S3. The scanned images and EDS results are shown in the figure below. Take the point target as one of the AgINPs in the figure. The test result shows that the mass fraction of the EDS in the Ag is 49.81wt%, and I is 50.19wt%, and in the mole fraction Ag is 53.87at%, and I is 46.13at %. Therefore, the synthesis ratio of AgINP is close to 1:1, which is consistent with the original measurement results in the article.


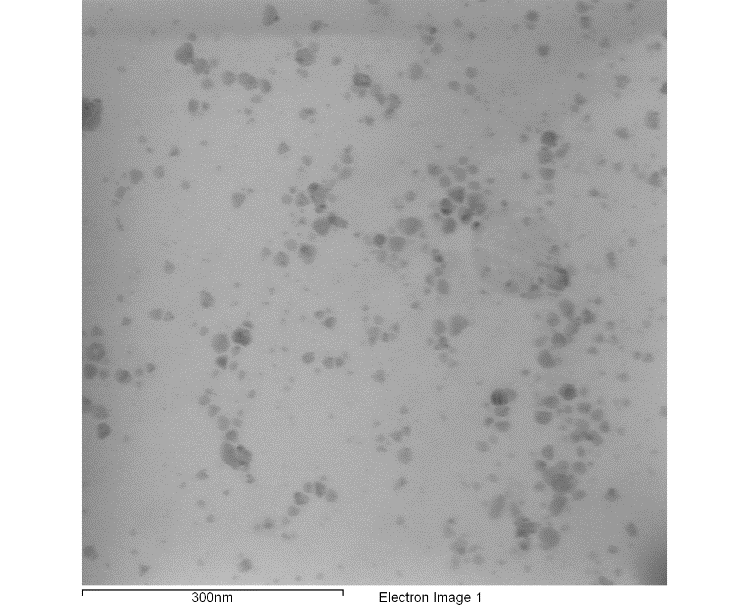

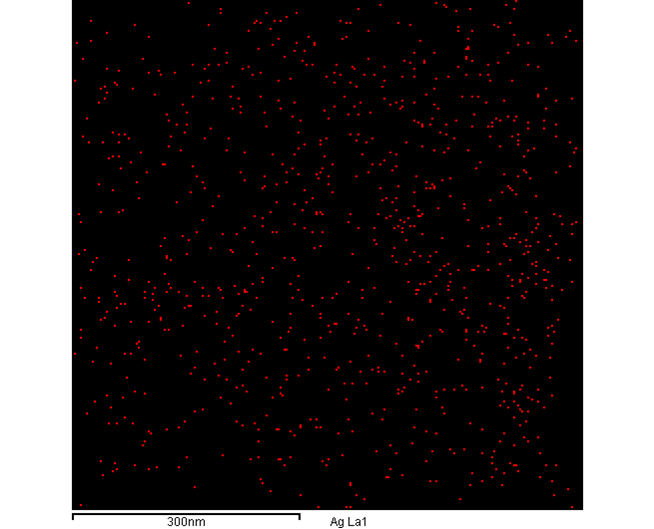


(a) (b)


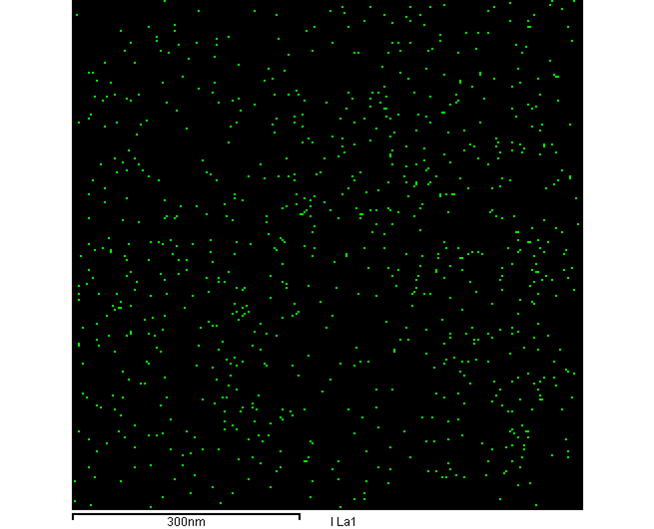

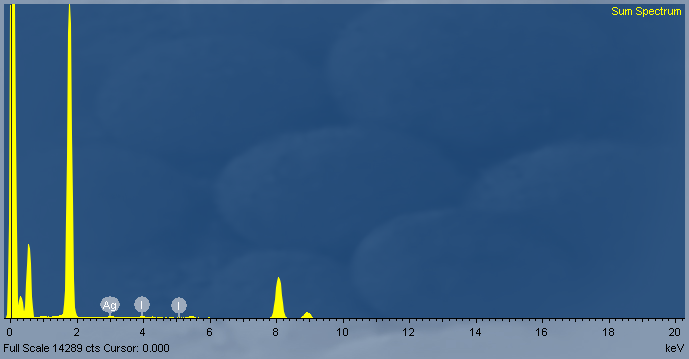


(c) (d)

Supplementary Figure S2 EDS mapping results (a)analysed image (b)silver element (red) (c)iodine element (green) and (d)EDS


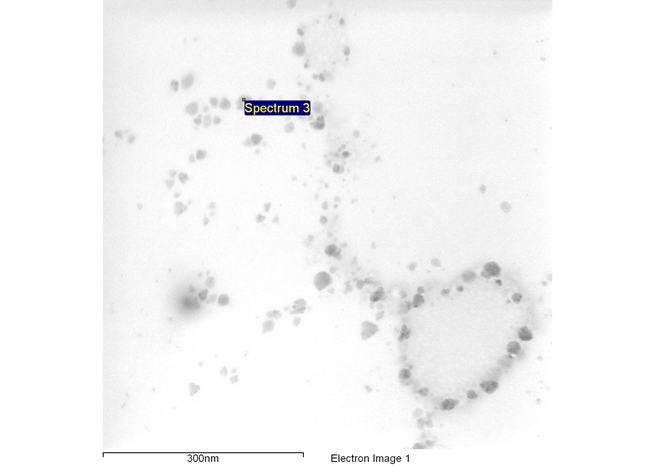

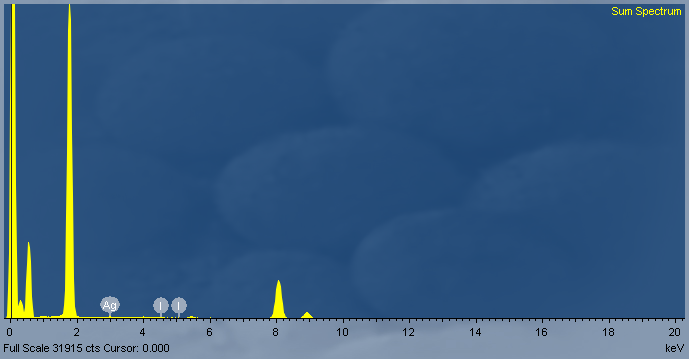


(a) (b)

Supplementary Figure S3 EDS scanning by taking points results (a)analysed image (b)EDS
